# Apples to apples: can differences in out-of-hospital cardiac arrest incidence and outcomes between Sweden and Ireland be explained by core Utstein variables?

**DOI:** 10.1186/s13049-018-0505-2

**Published:** 2018-05-03

**Authors:** Siobhán Masterson, Anneli Strömsöe, John Cullinan, Conor Deasy, Akke Vellinga

**Affiliations:** 1 Discipline of General Practice, National University of Ireland Galway, Distillery Road, Newcastle, Galway, Ireland; 2 Dalarna University, Falun, Sweden; 3 School of Business & Economics, National University of Ireland Galway, Galway, Ireland; 4 National Ambulance Service, Health Service Executive, Dublin, Ireland; 5 School of Medicine, National University of Ireland Galway, Galway, Ireland

**Keywords:** Out-of-hospital cardiac arrest, Utstein, incidence, outcomes, pre-hospital resuscitation

## Abstract

**Background:**

Variation in reported incidence and outcome based on aggregated data is a persistent feature of out-of-hospital cardiac arrest (OHCA) epidemiology.

**Objective:**

To investigate the extent to which patient-level analysis using core ‘Utstein’ variables explains inter-country variation between Sweden and the Republic of Ireland.

**Methods:**

A retrospective cross-sectional comparative study was performed, including all Swedish and Irish OHCA cases attended by Emergency Medical Services (EMS-attended OHCA) where resuscitation was attempted from 1st January 2012 to 31st December 2014. Incidence rates per 100,000 population were adjusted for age and gender. Two subgroups were extracted: (1) Utstein - adult patients, bystander-witnessed collapse, presumed medical aetiology, initial shockable rhythm and (2) Emergency Medical Service (EMS)-witnessed events. Multivariable logistic regression analysis was used to identify predictors of survival following multiple imputations of data.

**Results:**

Five thousand eight hundred eighty six Irish and 15,303 Swedish patients were included. Swedish patients were older than Irish patients (median age 71 vs. 66 years respectively). Adjusted incidence was significantly higher in Sweden compared to the Republic of Ireland (52.9 vs. 43.1 per 100,000 population per year). Proportionate survival in Sweden was greater for both subgroups and all age categories. Regression analysis of the Utstein subgroup predicted approximately 17% of variation in outcome, but there was a large unexplained ‘country effect’ for survival in favour of Sweden (OR 4.40 (95% CI 2.55–7.56)).

**Conclusions:**

Using patient level data, a proportion of inter-country variation was explained, but substantial variation was not explained by the core Utstein variables. Researchers and policy makers should be aware of the potential for unmeasured differences when comparing OHCA incidence and outcomes between countries.

**Electronic supplementary material:**

The online version of this article (10.1186/s13049-018-0505-2) contains supplementary material, which is available to authorized users.

## Key questions

- What is already known about this subject?
  There is variation in the reported incidence and outcomes from out-of-hospital cardiac arrest when aggregated data is compared
- What does this study add?
  This study quantifies the proportion of variation between countries that can be explained using patient level analysis that includes core Utstein variables
- How might this impact on clinical practice?

The significant potential for the presence of unmeasured differences between countries should be acknowledged, whether for research or for the development of national outcome targets.

## Background

There is international variation in the reported incidence and outcome from out-of-hospital cardiac arrest (OHCA). Chamberlain and Eisenberg stated that in order to compare outcomes between different systems of care, it is necessary to have “a comparator that enables areas of weakness to be defined and addressed whether it be at local, national and international level”. The Utstein criteria were developed for this reason, and identify patients based on a number of indicators [1]. International benchmarking is a highly desirable aspiration, and many notable studies and reviews have been carried out that compare the outcomes from OHCA across multiple countries and jurisdictions [2–5]. To ensure that comparison is informative, it is essential that data is collected for the same purpose, data definitions and collection methodologies are similar, and that the population covered is equally representative [6]. Assuring uniformity in OHCA data collection and reporting systems is essential, as differences in outcomes may well be attributable to differences in data availability and processing methodologies [7]. This aim of this study was therefore to investigate the extent to which patient-level analysis using core ‘Utstein’ variables explains variation in OHCA incidence and outcome between two countries, namely Sweden and the Republic of Ireland.

## Methods

### Aim and study design

We conducted a retrospective analysis of prospectively collected cohort of all Swedish and Irish cases of EMS-attended OHCA where resuscitation was attempted from 1st January 2012 to 31st December 2014.

### Data sources

Swedish OHCA registry data have been used to monitor national changes in OHCA incidence, management and outcome for more than 25 years [8]. Ireland’s OHCA registry was established in 2007, modelled on the Swedish registry, and has had comprehensive national data collection since 2012 [9]. Both registries are hosted by universities, operate in collaboration with ambulance services, and are publicly funded. Irish data is transcribed by an external data management company from ambulance patient care reports while Swedish practitioners enter event data directly onto a web-based template that is forwarded to the registry. Dispatch data and patient outcome data is available directly from the dispatch centre and receiving hospitals in both countries. The availability of a unique patient identifier in Sweden means that status of the patient at thirty days can be obtained from Statistics Sweden. Both countries have systematic missing case identification procedures. This is performed centrally in Ireland and at county level in Sweden. Similarly, regular data quality assurance is performed, using the original patient care report (Ireland) or medical journal (Sweden) to validate data entered. A full description of the data collection comparison is available as a supplementary table. During the study period, clinical practice guidelines for EMS in both countries complied with the 2010 ILCOR recommendations [10]. Irish and Swedish EMS practitioners are not required to start resuscitation in cases where definitive signs of death are present.

### Study setting

Sweden has a population of 9,995,153, occupying an area of 450,295 km^2^ [11]. Approximately 85% of the Swedish population lives in cities. Ambulance provision is governed at county and municipal level, and vehicles are primarily staffed by nurses and paramedics. In some cities, physicians may also be part of the ambulance crew [12]. Fire and police personnel are increasingly involved in providing a first response to OHCA in Sweden with public access defibrillators having been installed across the country [13].

The 2016 census showed that Ireland had a population of 4,757,976 [14]. Ireland is significantly smaller than Sweden (68,890 km^2^) with approximately 63% living in urban areas. Statutory Emergency Medical Services (EMS) are provided by the National Ambulance Service (NAS). In Dublin city, Dublin Fire Brigade (DFB) also provides the statutory response. Paramedic and advanced paramedics are deployed to suspected OHCA events. Emergency medical technicians may also be deployed as support crew or first responders to OHCA calls. Community first responder (CFR) volunteerism is becoming increasingly prominent in Ireland, with approximately 150 CFR schemes now established across the country. A more detailed description of the Irish EMS has been published elsewhere [9].

### Data processing

Register managers from both countries met to examine variables collected in both registries and identify which variables were comparable. Data were extracted from both registries, including original and derived variables. Variables with excessive missing values for either country (> 25%) were excluded from further analysis (available as Additional file 1).

Data were analysed using IBM SPSS Statistics v22.0© and STATA/IC 13.0 for Windows©. A subgroup was extracted based on the Utstein recommendations and included only adult patients with a bystander-witnessed collapse, with presumed medical aetiology and initial shockable rhythm [1]. A second subgroup of EMS-witnessed events was also extracted.

### Statistical analysis

Variables were categorised into patient and event characteristics, interventions and outcomes. Where significant differences in variables were observed, further analysis of variables by 5 year age groups was performed.

Sweden is divided into 21 administrative counties while the Republic of Ireland is composed of 34 (hereafter referred to as ‘admin areas’). For each admin area, crude incidence rates were calculated for all cases, both subgroups and for three age categories: children (under 18 years); adults (18–64 years); older people (65 years and over). Crude rates were adjusted to account for the proportion of the total population by gender in each age group at admin area level. Swedish population figures were derived from Statistics Sweden data and were averaged for the years 2012–2014 for each admin area [15]. Population estimates for the Republic of Ireland for each admin area were taken from the 2011 census [16]. An average country value was calculated from admin area values for each age and gender group with their 95% confidence intervals. The analysis of variance method (ANOVA) was used to compare average incidence rates. A *p*-value of < 0.05 was considered significant.

For key variables, missing data were imputed using a fully conditioned specification (FCS) or chained equations imputation model [17]. Imputation was performed separately for each country before data were merged for analysis.

Multivariable logistic regression analysis was used to identify predictors of the main outcome of interest (Discharge from hospital alive or alive at 30 days (Survival)). Models were estimated using original data (available as supplementary data) and imputed data. Potential explanatory variables were chosen based on previous literature and clinical relevance. Both ‘epinephrine’ and ‘mechanical CPR’ were initially included but were dropped due to insignificance. Interactions between the variable ‘Country’ and all other variables were checked. Due to non-linearity, the call-response interval variable was transformed into a binary variable for the regression analysis. Interactions which changed the Odds Ratios (ORs) for any of the main variables by more than 10% were included in the final model.

Model fit was assessed using imputed estimates adjusted R^2^, which were calculated using Harel’s method [18]. Calibration of individual imputed models was assessed using the Hosmer and Lemeshow *X*^*2*^ statistic (*p* > 0.05).

## Results

### Patient, event and intervention characteristics

A total of 5886 Irish and 15,303 Swedish patients were included in the analysis. The median test showed that Swedish patients were significantly older than Irish patients for all cases and in both subgroups (Table 1). Only gender was shown to have a similar distribution. All other variables showed differences between the countries. There were differences in the three categories of witness status, particularly the proportion of patients who had an EMS-witnessed arrest (Sweden – 15.2% vs. the Republic of Ireland – 5.9%, Table 1).

**Table 1:** Comparison of case characteristics between Republic of Ireland and Sweden – all Cases, Utstein and EMS-witnessed subgroups

	All cases		Utstein subgroup^c^		EMS-witnessed subgroup	
	Ireland	Sweden	Ireland	Sweden	Ireland	Sweden
Number of patients	(*n* = 5886)	(*n* = 15,303)	(*n* = 920)	(*n* = 2015)	(*n* = 335)	(*n* = 2211)
Patient and scene characteristics						
Median age in years (inter-quartile range)	66 (52–78)	71 (60–81)	65 (55–75)	69 (60–77)	69 (58–81)	74 (64–84)
Male (%)	67.9	66.6	78.8	82.3	208 (62.1)	1348 (61.0)
Incident occurred outside home (%)	32.7	30.1	51.6	59.1	174 (51.9)	1158 (53.7)
Presumed medical (%)	88.0	89.7	NA	NA	305 (91.0)	2086 (94.3)
Initial rhythm shockable (%)	23.7	23.7	NA	NA	107 (33.1)	496 (28.7)
Witness status (%)			NA	NA	NA	NA
Not witnessed (%)	40.5	33.7	NA	NA	NA	NA
Bystander (%)	53.5	51.1	NA	NA	NA	NA
Crew (%)	5.9	15.2	NA	NA	NA	NA
Interventions						
CPR before ambulance arrival (%)^a^	66.3	68.8	79.6	80.2	NA	NA
Who started CPR before ambulance arrival (%)						
Bystander, not dispatched (%)	60.7	56.9	44.3	52.4	NA	NA
Trained, may be dispatched by ambulance control (%)	33.5	12.8	31.9	9.9	NA	NA
Fire service (%)^b^	2.8	28.2	1.9	16.3	NA	NA
Police or fire and police (%)	2.1	3.0	1.5	1.6	NA	NA
Defibrillation before ambulance arrival (%)^a^	5.6	7.3	22.8	23.4	NA	NA
EMS call-response interval in minutes (median)^a^	13 (8–20)	10 (6–15)	12 (8–18)	8 (5–14)	NA	NA
Epinephrine (%)	63.8	80.1	65.7	80.4	58.1	66.3
Mechanical CPR (%)	4.6	35.9	4.9	41.1	7.8	25.8
Transported to hospital	53.8	60.9	75.7	84.2	74.9	83.4
Outcomes						
Any ROSC	23.2	32.8	45.9	54.9	42.3	45.1
ROSC at hospital arrival or arrived at hospital alive	16.9	24.4	37.1	49	32.1	35.4
Discharged alive from hospital	6.0	UA^c^	22.2	UA	16.8	UA
Alive at 30 days	UA	11.2	UA	31.7	UA	20.0
Discharged alive or alive at 30 days	6.0	11.2	22.2	31.7	16.8	20.0

*CPR* Cardiopulmonary Resuscitation, *EMS* Emergency Medical Services, *NA* Not applicable, *ROSC* Return of Spontaneous Circulation, *UA* Unavailable data

^a^For ‘All Cases’ this variable includes only cases NOT witnessed by EMS (Ireland *n* = 5342; Sweden *n* = 12,335)

^b^Fire Service’ includes all city and county fire services EXCEPT Dublin Fire Brigade

^c^The Utstein Subgroup includes patients who meet the following criteria – aged over 17 years, bystander-witnessed collapse, presumed medical aetiology, initial shockable rhythm

There were differences between countries in who provided CPR. ‘Trained, may be dispatched by ambulance control’ included members of the community who had training in CPR i.e. community first responders, off-duty paramedics, nurses, doctors, other clinical personnel. While it is known that many of these individuals were dispatched to the event by ambulance control, it was not possible to accurately determine that proportion. In the Republic of Ireland, this category accounted for 33.5% of providers of CPR before ambulance arrival compared to 12.8% in Sweden. Conversely, in Sweden 28.2% of CPR before ambulance arrival was provided by the fire service compared with 2.8% in the Republic of Ireland. A greater proportion of Swedish patients received defibrillation before ambulance arrival, though the actual percentages in both countries were small (5.6% and 7.3%), and the median EMS call-response interval was significantly shorter in Sweden.

### Differences in OHCA incidence and outcome between countries

The incidence of OHCA resuscitation attempts per 100,000 population per year was similar in both countries for the Utstein subgroup, despite the fact that the annual crude and adjusted incidence of OHCA was significantly higher in Sweden for all patients and for the EMS-witnessed subgroup (Table 2). This was also the case for all age categories for both genders, and for the EMS-witnessed subgroup.

**Table 2:** Incidence of out-of-hospital cardiac arrest resuscitation attempts per 100,000 population per year

	Ireland		Sweden	
Crude Incidence	Incidence per 100,000 population per year (95% CI)			
All Cases	43.1 (39.8–46.3)^*^		52.9 (47.7–58.1)^*^	
Utstein Subgroup	6.8 (6.1–7.4)		7.7 (6.5–8.9)	
EMS-witnessed	2.6 (2.0–3.1)^*^		8.1 (7.1–9.2)^*^	
Adjusted Incidence				
All Cases	42.3 (39.6–45.1)^*^		50.7 (46.1–55.2)^*^	
Age Categories	Male	Female	Male	Female
All Ages	28.7 (26.5–31.0)^**^	14.3 (13.1–15.5)^a^	35.5 (31.9–39.1)^**^	17.3 (15.4–19.2)^a^
U18	0.7 (0.6–0.9)	0.5 (0.3–0.7)	0.7 (0.4–1.0)	0.4 (0.2–0.5)
18–64	13.4 (12.2–14.6)	5.2 (4.6–5.8)	12.0 (10.8–13.3)	5.1 (4.4–5.8)
65+	14.1 (13.0–15.3)^**^	8.4 (7.6–9.3)^a^	21.0 (19.2–22.8)^**^	11.4 (10.2–12.6)^a^

*EMS* Emergency Medical Services

^*^ Higher incidence in Sweden (*p* < =0.05)

^**^ Higher incidence in Swedish males than Irish males (*p* < =0.05);

^a^ Higher incidence in Swedish females than Irish females (*p* < =0.05)

The proportion of surviving patients was consistently higher in Sweden for all patients, the Utstein subgroup and the EMS-witnessed subgroup (Table 3). This significant differential in survival was persistent across both genders and all age categories.

**Table 3:** Proportion of surviving patients categorised by country, gender and age group

	Ireland		Sweden	
	*n* (%)			
All cases	350 (6.0)^*^		1686 (11.2)^*^	
Utstein Subgroup	200 (22.2)^*^		634 (31.7)^*^	
EMS-witnessed	56 (16.8)^*^		436 (20.0)^*^	
Age Categories	Male	Female	Male	Female
All ages	283 (7.2)^**^	67 (3.6)^a^	1276 (12.8)^**^	410 (8.1)^a^
U18	9 (8.8)^**^	2 (3.2)^a^	35 (24.3)^**^	13 (16.0)^a^
18–64	164 (9.3)^**^	35 (5.8)^a^	614 (18.7)^**^	150 (11.7)^a^
65+	103 (5.3)^**^	30 (2.6)^a^	616 (9.8)^**^	244 (6.8)^a^

*EMS* Emergency Medical Services

^*^ Higher incidence in Sweden (*p* < =0.05)

^**^ Higher incidence in Swedish males than Irish males (*p* < =0.05)

^a^ Higher incidence in Swedish females than Irish females (*p* < =0.05)

### Extent of unmeasured variation between countries

Variables independently associated with improved ORs for Survival were younger age, collapse at a location other than home, CPR and defibrillation before ambulance arrival and a shorter EMS call-response interval (Table 4). Gender was not significantly associated with Survival at a 5% level after controlling for interactions at country level. After adjustment for other variables and inclusion of interaction terms, the OR for Survival in Sweden was 4.40 (95% CI 2.55–7.56). Interactions included in the final model were country*gender and country*home location. There was no significant difference between results obtained from original or imputed data. The overall proportion of variation in Survival that is accounted for in the final model was relatively small (adjusted R^2^ 17%).

**Table 4:** Multivariable logistic regression analysis for the outcome survival in the Utstein subgroup (adult, bystander-witnessed, initial rhythm, shockable, presumed medical aetiology)

	Survival
	Adjusted Odds Ratio (95% CI)
Sweden	4.28 (2.51–7.30)
Age (in years)	0.97 (0.96–0.97)
Male	1.60 (1.02–2.52)
Not at home	3.05 (2.16–4.30)
CPR before ambulance arrival	1.65 (1.30–2.10)
Defibrillation attempted before ambulance arrival	1.40 (1.13–2.74)
Call Response Interval 5 min or less	1.97 (1.61–2.40)
*Model Fit*	
Nagelkerke R^2^ adjusted	0.17

Hosmer and Lemeshow Test not significant for any imputations

## Discussion

This patient-level analysis of 3 years of data from two well established national registries shows that the incidence of attempted resuscitation is similar for the Utstein subgroup in both countries, but that percentage survival is greater in Sweden than in the Republic of Ireland overall, for all age categories and both subgroups. Even when data from two countries has been collected using similar methods and rationale, the reasons for inter-country differences in outcome are not fully explained by the core Utstein variables used in this study.

Our study highlights that differences in OHCA outcomes between countries are not solely down to differences in patient age and gender profile or pre-hospital interventions. By using patient level data, this analysis serves to quantify the degree of variation that can be explained by inter-country comparison in a way that cannot be achieved with aggregate outcome data. The critical value of OHCA data collection is that it can focus national efforts on improving national outcomes [19, 20]. In the latest revision of the Utstein dataset, Perkins et al. advised on a range of core and supplemental OHCA elements that are likely to help explain a larger proportion of inter-country outcome variation, including pre-existing co-morbidities and in-hospital treatments and interventions [21]. Implementation or improved systematic collection of these data elements is likely to explain substantial variation in outcome within and between countries.

There are clearly differences in the patient and intervention characteristics in both countries. On average, Swedish patients are older in all cases and in both subgroups, and the higher overall incidence of OHCA resuscitation is largely accounted for by the greater proportion of older OHCA patients in Sweden (Table 2). The explanation for this difference in age profile may lie in cultural attitudes and expectations surrounding death and morbidity in both countries. A survey of public attitudes to resuscitation in older people has not been previously carried out, but may help explain the significant difference in resuscitation incidence in this age category.

As also shown in Table 2, the incidence of Utstein subgroup cases is similar in both countries. This is likely to be largely driven by the fact that similar proportions of patients had an initial recorded shockable rhythm (23.7%). The ‘three phase model’ of cardiac arrest suggests that most patients will deteriorate into an asystole within 5 min without intervention [22]. Considering the significantly shorter median EMS call response-interval in Sweden, it may have been expected that the proportion of Swedish patients with an initial recorded shockable rhythm would be greater than in the Republic of Ireland. One explanation may be the higher proportion of older people in the Swedish OHCA resuscitation population, as older people have been found to have a lower incidence of initial shockable rhythm [23]. Additionally, a decline in the proportion of patients with an initial shockable rhythm has previously been observed in Sweden, despite efforts to improve call-to-shock times [24]. It has been proposed that this decline may be due to a reduction in untreated ischaemic heart disease (IHD) in the Swedish population and that the proportion of cases with cardiac aetiology is less than presumed [25]. Diagnosis of IHD continues to increase the Republic of Ireland and what proportion of this increase is due to increasing prevalence or improved detection is unclear [26]. Both registries primarily rely on the clinical impression formed by the attending ambulance crew to determine the aetiology of arrest. Previous work on validation of aetiology in paediatric OHCA has shown the potential value of adding coronial data to an OHCA registry [27]. It is suggested that inclusion of coronial data in the Swedish and Irish registries may assure the validity of data on aetiology and ensure realistic expectations for the proportion of cases with an initial shockable rhythm.

The proportion of CPR provided by those who were ‘trained, may be dispatched by ambulance control’ in the Republic of Ireland is encouraging (Table 1). The Republic of Ireland already has an active and growing Community First Responder (CFR) network. While there is evidence that trained first responders can contribute to survival, the best model of CFR is not yet determined [28]. The fire service plays a greater role in the provision of CPR in Sweden, suggesting there is potential for Irish Fire Services to participate more often in the OHCA response. It should be noted that – despite the fact that that dual dispatch of ambulance and fire services in Sweden has been shown to have the greatest effect on response intervals in rural Swedish areas – survival benefit was most significant in densely populated areas [29]. This suggests that there is a response interval beyond which any form of dual dispatch may not be of additional benefit to ambulance dispatch only.

Proportionate survival from OHCA is greater in Sweden for all patients, both subgroups and all age categories (Table 3). Patients who collapse in the presence of EMS are likely to receive good quality CPR and rapid defibrillation, which in turn is more likely to be immediately effective if performed soon after collapse [30]. This is borne out in the relatively high proportion of survival in this subgroup in both countries, and partially explains the higher overall percentage survival in Sweden.

The multivariable logistic regression model of survival explains – at best – 17% of variation between countries, and includes a large ‘country effect’ in favour or Sweden that is not explained by the predictor variables (Table 4). Rather than suggesting that the chances of patients in the Utstein group surviving an OHCA are over four times greater in Sweden than in the Republic of Ireland, this result points to the large proportion of variation which is not explained by our Utstein predictor variables. The implication is that while improving the availability of important outcome predictors such as bystander CPR and defibrillation, and reducing EMS call response intervals is likely to increase survival in the Republic of Ireland, these measures alone are unlikely to achieve parity of outcomes with Sweden.

### Limitations

Simplified coding was applied to many variables in order to facilitate systematic registry recording and inter-country analysis. Most notably, we created the variable ‘Survival’ using the different outcome measures used in Sweden and the Republic of Ireland. In the Republic of Ireland, the primary outcome is ‘discharged alive from hospital’. Patients are not included as OHCA survivors until discharged, regardless of the length of their acute hospital stay. In Sweden survivors are classified as those who are alive 30 days or more after the event, even if the patient has not been discharged from an acute facility. While it is possible that Irish patients who are discharged alive may not survive to 30 days, it is also possible that Swedish patients may remain as in-patients for 30 days or more. Both outcome measures have been used interchangeably in other national comparative studies, and the use of either outcome measure has been recommended in the Utstein guidelines [21]. In general, it is not usual for studies to report both these outcomes. In cases where both outcomes have been reported, there is negligible difference in the number of surviving patients [31, 32].

While the proportion of patients who had defibrillation attempted before ambulance arrival is similar for the Utstein subgroup in both countries, 14.8% of Swedish cases had missing data for this variable. Using the original data the adjusted OR for this variable in the logistic regression analysis was 1.41 (95% CI 1.11–1.78) compared to 1.40 (95%CI 1.13–2.74) using imputed data.

## Conclusions

The ability to compare OHCA incidence and outcomes across countries and systems is essential to establishing international benchmarking. The use of patient-level data have highlighted the proportion of variation outside of the well-known predictors of OHCA outcome, something that is not possible in comparative studies that rely on aggregated data. We believe the approach used in this study is transferable to other comparative studies of OHCA national incidence and outcome, and that such an approach will improve the validity and value of inter-country comparison, whether for research or for the development of national outcome targets.

## Additional file

Additional file 1: **Table S1.** Comparison of Irish and Swedish OHCA resuscitation registries, **Table S2.** Missing data items, **Table S3.** Logistic regression analysis for the outcome survival in the Utstein subgroup using original data (adult, bystander-witnessed, initial rhythm, shockable, presumed medical aetiology). (PDF 95 kb)
