# Supplementary material for: Apples to apples: can differences in out-of-hospital cardiac arrest incidence and outcomes between Sweden and Ireland be explained by core Utstein variables?
Source: Scand J Trauma Resusc Emerg Med. 2018 May 3;26:37. doi: 10.1186/s13049-018-0505-2 (PMC5934807; doi:10.1186/s13049-018-0505-2)
Supplement: Supplementary file 1 — Table S1. Comparison of Irish and Swedish OHCA resuscitation registries, Table S2. Missing data items, Table S3. Logistic regression analysis for the outcome survival in the Utstein subgroup using original data (adult, bystander-witnessed, initial rhythm, shockable, presumed medical aetiology). (PDF 95 kb) [file 13049_2018_505_MOESM1_ESM.pdf]

**Table S1** Comparison of Irish and Swedish OHCA resuscitation registries

| | Ireland | Sweden |
|---|---|---|
| **Managing Organisation** | OHCAR is based in the HSE North West Department of Public Health and is carried out in collaboration with the National Ambulance Service, National University Institute Galway and the Pre-Hospital Emergency Care Council (PHECC). The OHCAR Steering Group is responsible for directing the project and includes representatives from each organisation. | The OHCA register is based in Gothenburg, the West Coast in Sweden and collaborates with the Emergency Ambulance Services (EMS) in all 21 counties. The OHCA Steering Group members are from several counties in Sweden but the there is a working group situated in Gothenburg. |
| **Funding** | OHCAR is funded by the National Ambulance Service and the Pre-Hospital Emergency Care Council | The register is funded by The Swedish Association of Local Authorities and Regions |
| **Patient and Event Variables** | In Ireland, statutory Emergency Medical Services use a standardised Patient Care Report (PCR) which contains a section dedicated to data collection for OHCAR. Specially designed OHCA envelopes have also been provided to each ambulance station. In the event of OHCA, practitioners place completed PCRs in OHCA envelopes. On a monthly or fortnightly basis, envelopes are collected together with all PCRs from each station. All PCRs are scanned and stored digitally and cases in OHCA envelopes are given priority in the scanning process to facilitate OHCAR. OHCAR variables are manually entered onto an electronic database. This database is then forwarded to OHCAR together with a scanned copy of each PCR for case-by-case validation. | There is an standard web template which will be documented in connection to a treated OHCA. The EMS crew is responsible for filling in the web template. All data is collected in a database. |
| **Time variables** | Dispatch data (i.e. time variables) is collected directly from the ambulance dispatch centre by registry staff | The time variables are availible from the dispatch center and from the EMS medical journal. |
| **Outcome data** | Outcome data is requested by registry staff from receiving hospitals | Outcome data is availible from medical journals from EMS and in-hospital and also from Statistics Sweden. |
| **How are missing cases identified?** | Cases that are not placed in OHCA envelopes are not processed through the OHCAR data collection system and must be identified separately. Missing case identification is performed on a monthly basis and repeated on an annual basis to capture delayed reports. First, a search of the digital scanning archive is performed based on the 'Chief Complaint' field in the PCR using the word 'arrest'. Reported cases are excluded from the results and then the digital scan of the PCR associated with each call found is viewed. Missed OHCAR cases are identified and captured during the viewing process. Next, emergency control data is filtered to identify all calls with an AMPDS© designation of 'ECHO' at the time of resource deployment. A further seventeen 'DELTA' codes that may signify arrest occurred are included in the filter. Reported calls are then excluded from the filtered list. PCRs on the filtered list are then viewed and remaining unreported OHCAR cases are identified. | The EMS crew makes regular retrospective observations with the aim of searching undocumented OHCA. The searching procedure is performed by a digital searching programme, and manually searching if medical journals have been documented in papers. Missed OHCA cases are identified and imported to the database. Missed OHCAs are labelled in order to identify them as retrospective data. |

**Table S1** Comparison of Irish and Swedish OHCA resuscitation registries (continued)

| | Sweden | Ireland |
|---|---|---|
| **How is data quality assured in the registry?** | Data is received by OHCAR in an electronic database together with a scanned copy of each PCR. Each electronic entry is checked by OHCAR staff against the data in the PCR to ensure accuracy of manual data entry. Checked data is then forwarded to the OHCAR manager who performs a random check of cases before finally adding data to the master OHCAR database. For cases that are identified through the missing case identification process, data is extracted from the scanned PCR by OHCAR staff and manually entered onto an electronic database by OHCAR staff. The 'missing' database is then forwarded to the OHCAR manager, who validates each entry using the corresponding scanned PCR. Once validation of the missing cases is complete, they are added to the master OHCAR database. | Registry data is compared to documented data in medical journals using variables such as incidence, place OHCA occurred, treatment and survival. |

**Table S2** Missing data items

| Variable Information | Variable Type* | Ireland | Sweden |
|---|---|---|---|
| | | **Missing Data n(%)** | |
| Was OHCA identified by ambulance control at time of dispatch | Original** | 270 (4.6) | 15303 (100.0) |
| Was dispatch assisted CPR offered | Original** | 5886 (100.0) | 8386 (54.8) |
| Age | Original | 171 (2.9) | 484 (3.2) |
| Gender | Original | 6 (0.1) | 3 (0.0) |
| Who witnessed collapse | Original | 209 (3.6) | 757 (4.9) |
| OHCA location | Original | 40 (0.7) | 322 (2.1) |
| Home or not home location of collapse | Recode | 40 (0.7) | 322 (2.1) |
| CPR before ambulance arrival | Original | 197 (3.3) | 803 (5.2) |
| Who started CPR | Derived | 197 (3.3) | 842 (5.5) |
| Defibrillation before ambulance arrival | Recode | 172 (2.9) | 2973 (19.4) |
| First monitored rhythm | Original | 234 (4.0) | 1555 (10.2) |
| First monitored rhythm shockable or nonshockable | Recode | 234 (4.0) | 1555 (2.5) |
| Presumed aetiology | Original | 0 (0.0) | 0 (0.0) |
| Medical or non medical aetiology | Recode | 0 (0.0) | 0 (0.0) |
| Call-Response interval*** | Original | 810 (15.2) | 1030 (8.4) |
| Call to defibrillation interval**** | Original** | 811 (39.1) | 801 (15.0) |
| Epinephrine administered | Original | 54 (0.9) | 314 (2.1) |
| Airway control type | Original** | 505 (8.6) | 9868 (64.5) |
| Mechanical CPR | Original | 343 (5.8) | 1938 (12.7) |
| Transported to hospital | Original | 1 (0.0) | 118 (0.6) |
| Any ROSC | Original | 192 (3.3) | 892 (5.8) |
| ROSC or alive on arrival at hospital | Derived | 275 (4.7) | 240 (1.6) |
| Discharged alive or alive at 30 days | Derived | 64 (1.1) | 289 (1.9) |
| CPC at discharge from hospital***** | Original** | 80 (22.9) | 795 (47.2) |

*Original (taken directly from national register), Derived (variables or variable lists from national register combined to create single study variable or similar options for both registries), Recode (number of variables options reduced to faciliate analysis)
**Variable excluded from study due to excess missing data in either or both countries
***Not EMS-witnessed cases (Ireland n=5,342; Sweden n=12,335)
****For cases where defib attempted (Ireland n=2,072 ; Sweden n=5,327)
*****For cases where survival to discharge or 30 days confirmed (Ireland = 350; Sweden = 1,686)

**Table S3** Logistic regression analysis for the outcome survival in the Utstein subgroup using original data (adult, bystander-witnessed, initial rhythm, shockable, presumed medical aetiology)

| | **Survival** |
|---|---|
| | Adjusted Odds Ratio (95% CI) |
| Sweden | 3.60 (1.95 -6.68) |
| Age (in years) | 0.96 (0.96-0.97) |
| Male | 1.79 (1.00-3.23) |
| Not at home | 2.41 (1.96-2.97) |
| CPR before ambulance arrival | 1.40 (1.06-1.84) |
| Defibrillation attempted before ambulance arrival | 1.40 (1.78-2.86) |
| Call Response Interval 5 minutes or less | 2.25 (1.78-2.86) |
| *Model Fit* | |
| Nagelkerke $R^2$ adjusted | 0.17 |
| Hosmer and Lemeshow Test not significant for any imputations | |
